# Supplementary figures and images for: Development of dual enzyme responsive molecular AND logic gate
Source: Turk J Chem. 2021 Dec 24;46(2):567–74. doi: 10.3906/kim-2111-19 (PMC10734702; doi:10.3906/kim-2111-19)

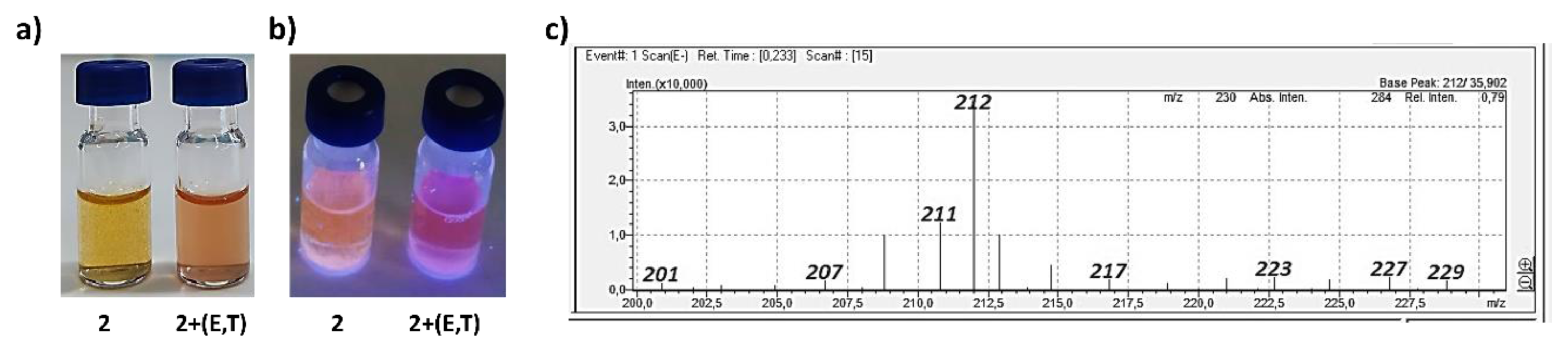

Supplement: Figure S1 — Compound 2 (100 μM) in PBS buffer (pH 7.4) under daylight (a) and under UV light (b). Compound 2 is incubated with esterase (2.5 U/ml) and tyrosinase (100 U/ml) enzymes for 30 min in right samples of each photo. Low resolution mass spectra obtained by LCMS analysis of enzyme treated sample (c) proves the formation of resorufin with m/z of 212 corresponding to (M-H)−. [file turkjchem-46-2-567s1.tif]

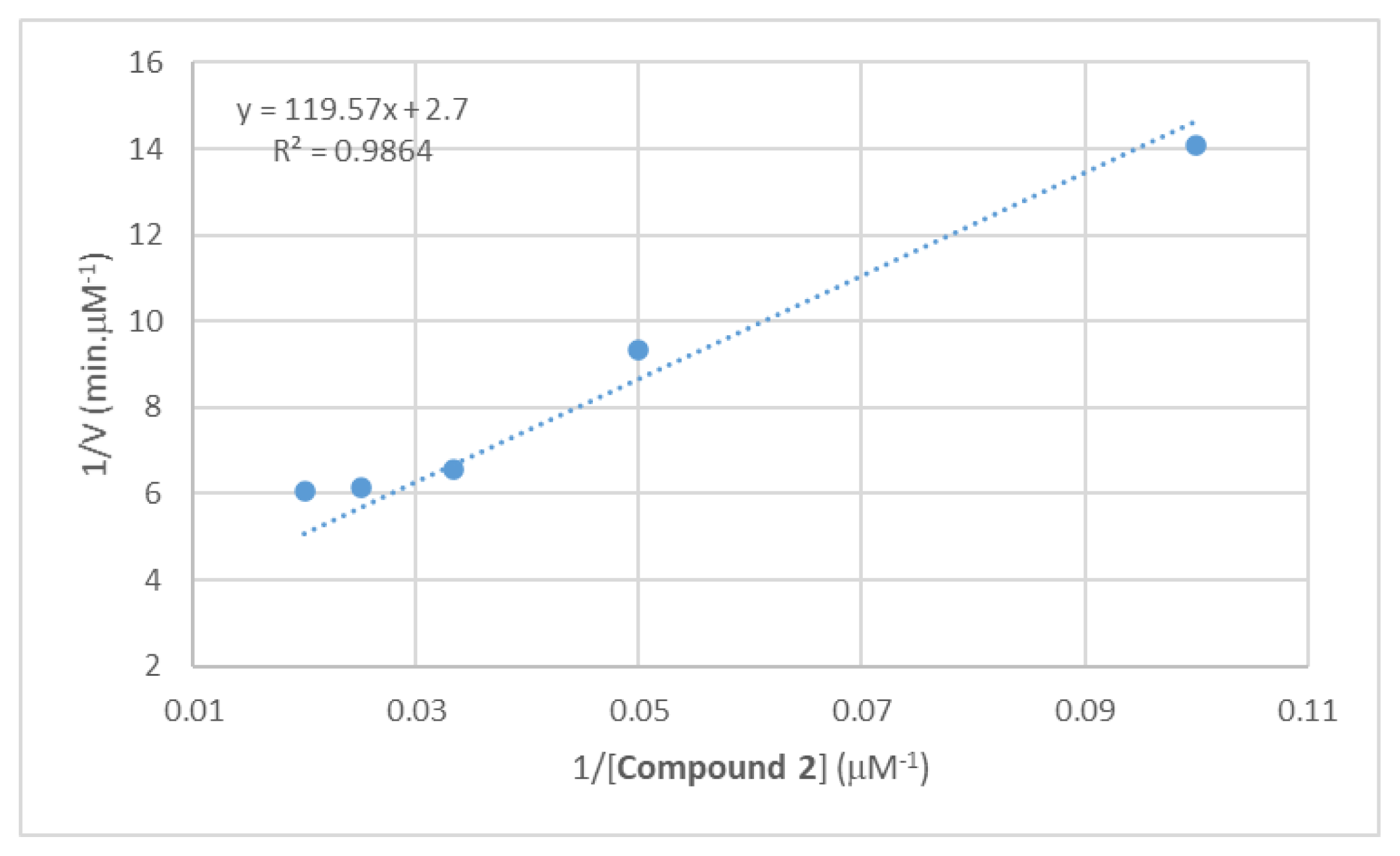

Supplement: Figure S2 — Lineweaver-Burke plot for conversion rate of compound 2 (10–50 μM) in the presence of 3 U/ml tyrosinase enzyme in PBS buffer, at 37°C. [file turkjchem-46-2-567s2.tif]

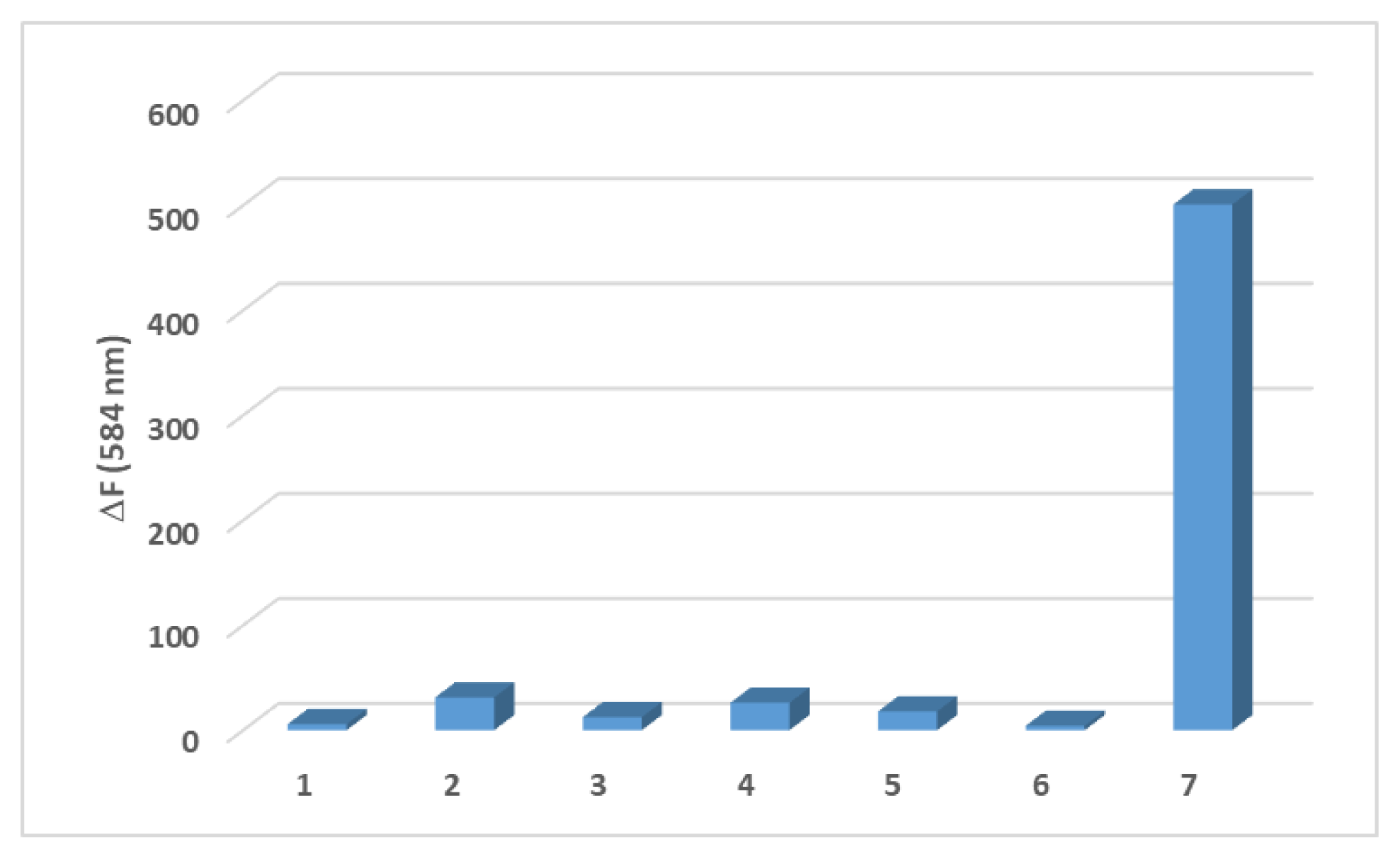

Supplement: Figure S3 — Selectivity of the compound 2 towards various different analytes. Samples contains (1) 100 μM BSA; (2) 1mM GSH; (3) 1 mM sodium ascorbate; (4) 100 μM H2O2; (5) 100 μM KO2; (6) 100 μM TBHP; (7) 25U/ml tyrosinase and 2.5U/ml esterase enzymes. Excitation wavelength is 550 nm. [file turkjchem-46-2-567s3.tif]

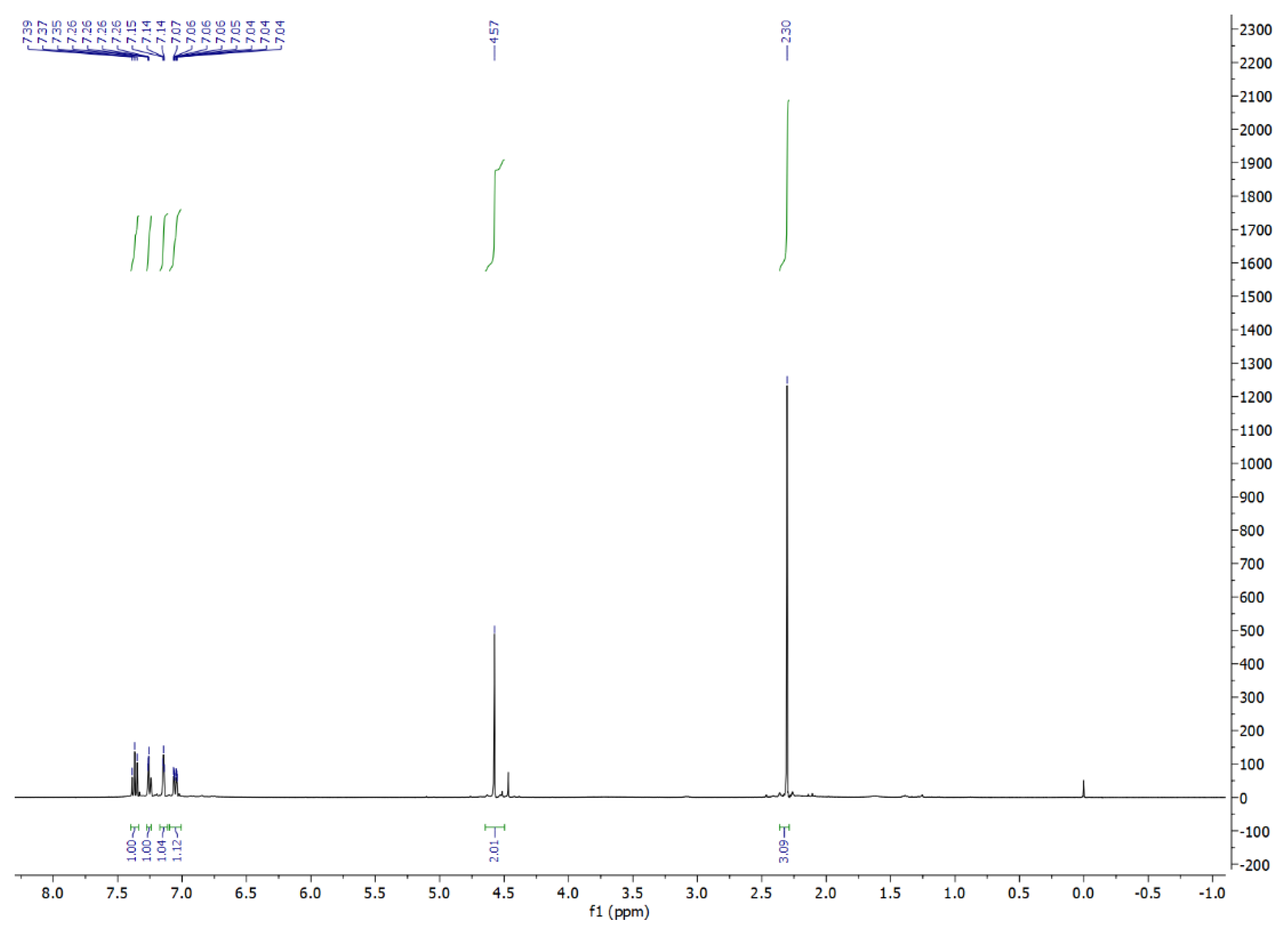

Supplement: Figure S4 — 1H NMR spectrum of compound 1 (400 MHz, CDCl3) [file turkjchem-46-2-567s4.tif]

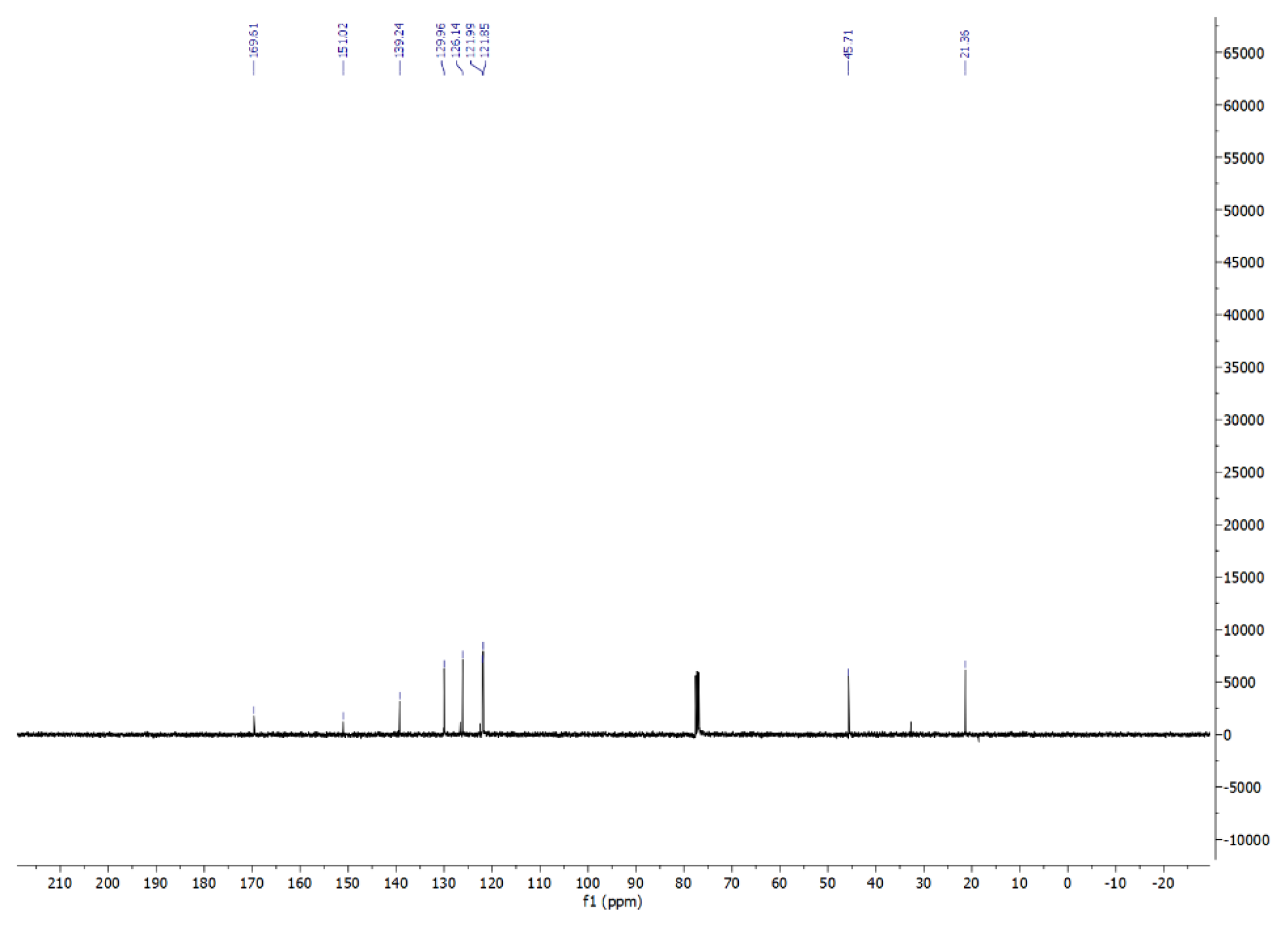

Supplement: Figure S5 — 13C NMR spectrum of compound 1 (400 MHz, CDCl3) [file turkjchem-46-2-567s5.tif]

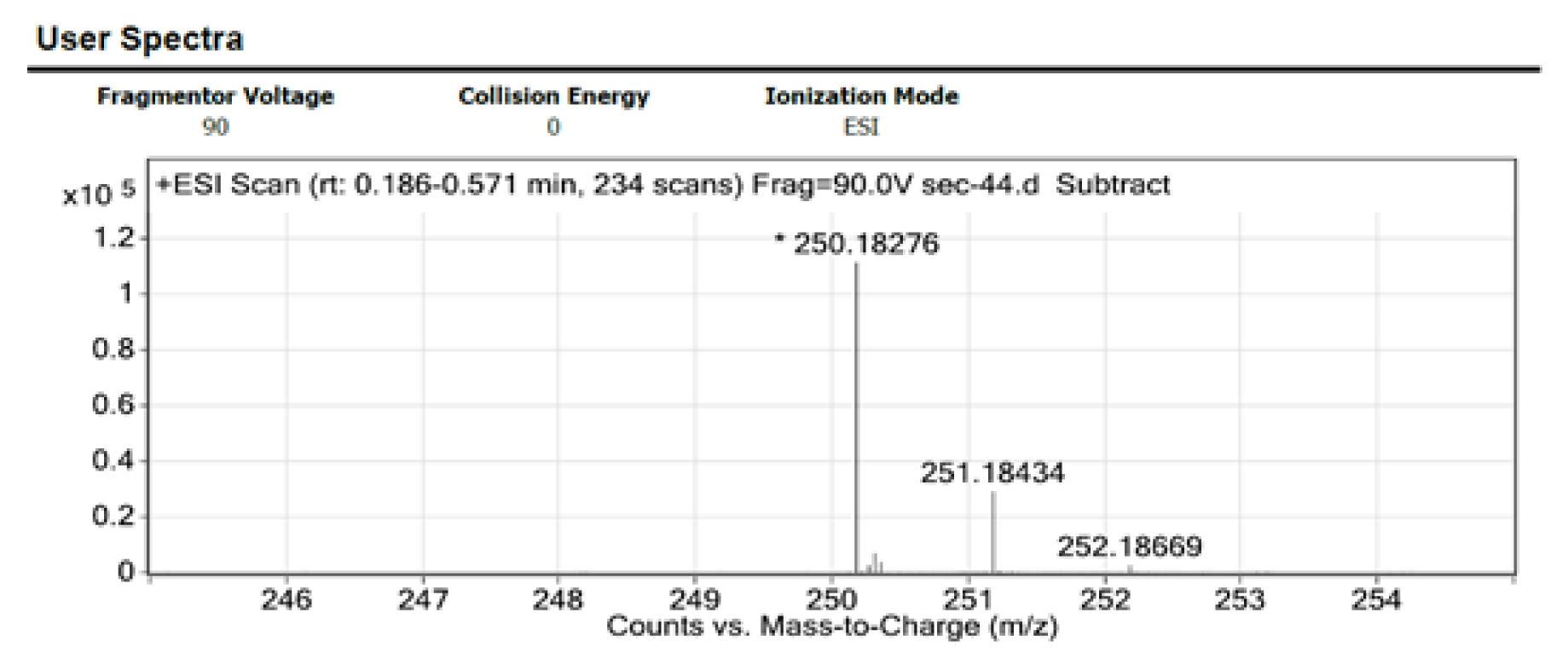

Supplement: Figure S6 — HRMS (ESI) spectrum of compound 1 [file turkjchem-46-2-567s6.tif]

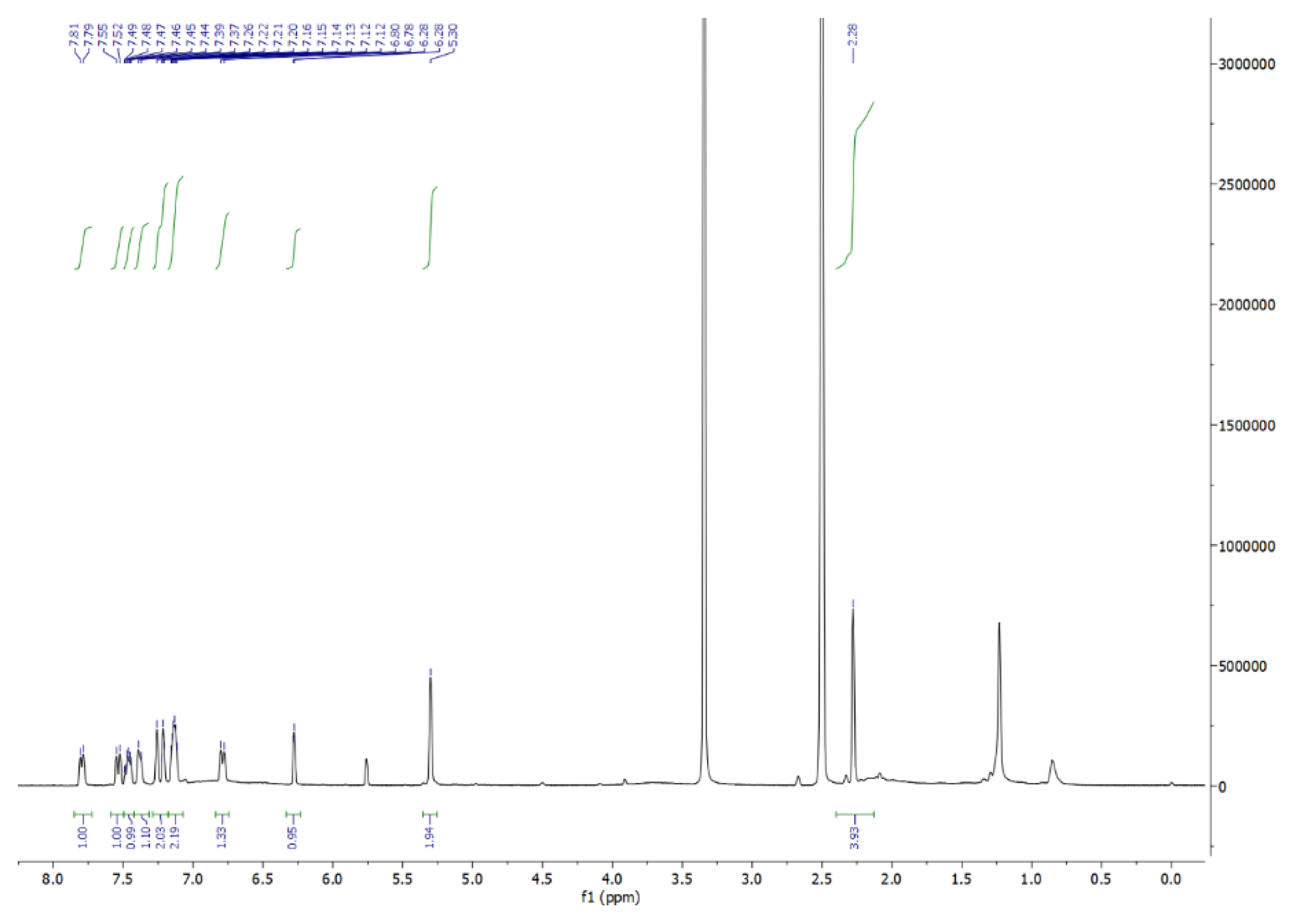

Supplement: Figure S7 — 1H NMR spectrum of compound 2 (400 MHz, DMSO-d6) [file turkjchem-46-2-567s7.tif]

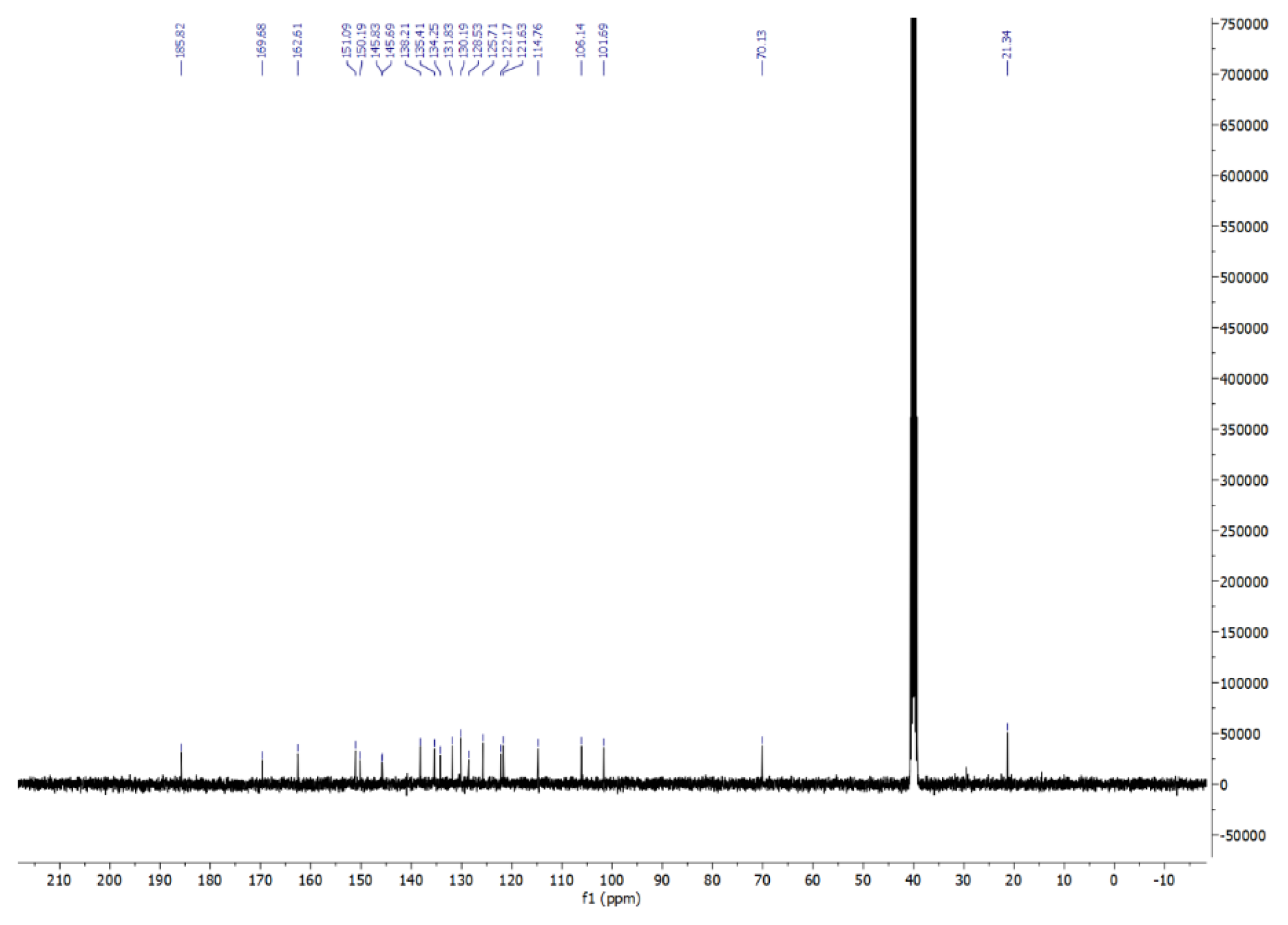

Supplement: Figure S8 — 13C NMR spectrum of compound 2 (400 MHz, DMSO-d6) [file turkjchem-46-2-567s8.tif]
